# Spontaneous arthritis and ankylosis in male DBA/1 mice: further evidence for a role of behavioral factors in “stress-induced arthritis”

**DOI:** 10.1186/1480-9222-14-10

**Published:** 2012-12-19

**Authors:** Kirsten Braem, Shea Carter, Rik J Lories

**Affiliations:** 1 Tissue Homeostasis and Disease, Skeletal Biology and Engineering Center, Department of Development and Regeneration, KU Leuven, Leuven, Belgium; 2 Division of Rheumatology, University Hospitals Leuven, Leuven, Belgium

**Keywords:** Spondyloarthritis, Spontaneous arthritis, Behavioral factors, Ankylosis

## Abstract

**Background:**

Ageing male DBA/1 mice spontaneously develop arthritis in the hind paws. We and others have demonstrated that this model shares striking features with human spondyloarthritis, in particular entheseal involvement, progressive ankylosis but also dactylitis. Here, we report on our recent experience with this model highlighting how changes in the animal facility affect the development of the disease.

**Findings:**

Ageing male DBA/1 mice from different litters were caged together (6 mice per cage) at the age of 10 weeks. The mice were checked twice a week for clinical signs of arthritis. Disease severity was assessed in further detail post-mortem by scoring for histomorphological characteristics. DBA/1 mice spontaneously develop macroscopically detectable arthritis, presenting as joint swelling or toe stiffness. Standard settings with open cages lead to an almost 100% incidence by the age of 26 weeks. The introduction of larger cages and filter tops reducing exposure to other cages dramatically affected incidence. Other negative factors include excess bedding material reducing the impact of walking and running. Switching back to the original conditions resulted again in a high incidence, further optimized by sensory exposure to female mice. We also showed that the related DBA/2 strain is sensitive to the disease.

**Conclusions:**

Changing environmental factors in the housing conditions of DBA/1 mice severely affects the spontaneous development of arthritis. This points out that the model is very sensitive to external stress and sensory factors that are likely affecting the behavior of the male mice and that the model needs to be optimized in different situations.

## Findings

Spondyloarthritis (SpA) is a group of common inflammatory and chronic joint diseases [1,2]. The clinical spectrum of SpA includes various diagnostic entities that share clinical, genetic and pathological characteristics. Ankylosing spondylitis (AS) is the prototype and best-known entity of SpA. The disease cluster is primarily characterized by inflammation and new bone formation in the axial skeleton and the joints. Both processes contribute to symptoms and can result in loss of function and disability [1,3]. Biopsies from spinal lesions are difficult to obtain. Consequently, SpA research has been hindered by limited availability of tissue samples from patients. Therefore, insights into molecular and cellular disease mechanisms are largely based on data obtained in different animal models. Although different mouse and rat models of SpA exist, none of these exactly mimics human disease. Our laboratory uses a spontaneous mouse model of arthritis in aging male DBA/1 mice (SpAD) [4-6] and here we report our recent experience with this model, highlighting how changes in the housing conditions can affect the disease course (Table 1) and demonstrating that the related strain DBA/2 is also sensitive to the disease. 

**Table 1:** Changed environmental factors in animal housing

	**Before**	**After**
Cage surface	335 cm^2^	370 cm^2^
Filter top	No	Yes
Bedding material	Low amount	Excess

### Spontaneous arthritis in male DBA/1 mice (SpAD): description of the model

**Figure 1:**
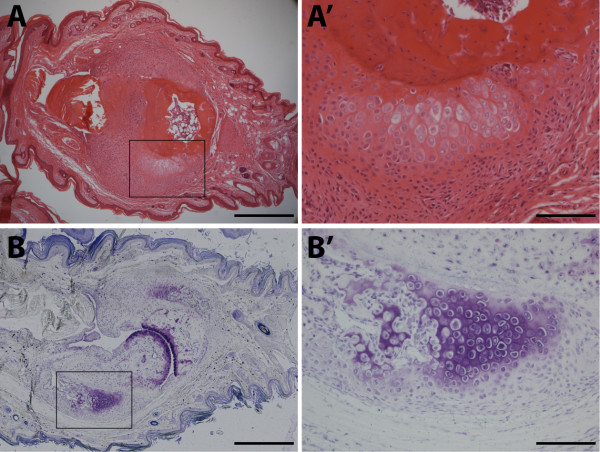
**Entheseal endochondral bone formation in male DBA**/**1 paws.** (**A**) H&E staining of a DBA/1 hind paw showing new cartilage and bone formation which originates from the enthesis. In (**B**), Toluidine blue staining of chondrogenic differentiation which occurs on both the proximal and distal side of the joint, resulting in ankylosis and loss of function of the joint. **A’** and **B’** showing higher magnifications of boxed areas in **A** and **B**, respectively. Scale bars: 500 μm in **A** and **B**, 125 μm in **A’** and **B’**.

**Figure 2:**
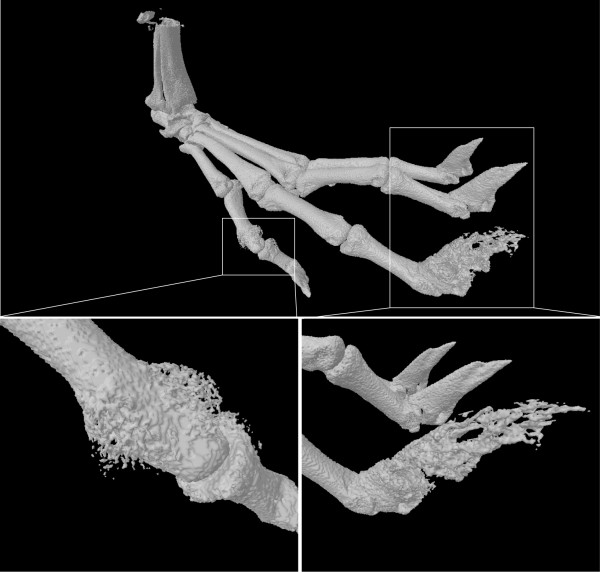
**Bone formation in the interphalangeal joint of male DBA**/**1 hind paw as assessed by X**-**ray micro**-**computed tomography** (**μCT**)**.** μCT imaging of the hindpaw forefeet of a 17 week old DBA/1 mouse shows new bone formation in the proximal interphalangeal joint of the 5th toe and late stage onychoperiostitis of the 4th toe. The mouse bones were visualized *ex vivo* using Skyscan 1172 μCT system and related SkyScan software (Kontich, Belgium). The scanning parameters were 5 μm pixel size, 50 kV, 200 uA, 0.5 mm Al filter. Data images acquired at multiple viewing angles were reconstructed to generate a full 3D representation of the hind paw.

The murine ankylosing enthesitis model has become one of the most-studied experimental models for AS and SpA. Ageing male DBA/1 mice spontaneously develop arthritis in the hind paws after grouped caging from the age of 12 weeks onwards [5,7]. It is essential to group mice from different litters for arthritis to develop. The model is characterized by a short bout of acute inflammation [5] followed by entheseal endochondral bone formation, especially in the interphalangeal joints of the hind paws. Microscopic images demonstrate proliferation and condensation of spindle shaped cells in close connection to an enthesis, the insertion site of tendons and ligaments onto the underlying bone (Figure 1). Within the cell mass, chondrogenic differentiation is initiated typically in contact with the underlying bone. A full cascade of endochondral bone formation is recapitulated with progressive differentiation of the chondrocytes towards hypertrophy and subsequent replacement of the cartilage by new bone, eventually leading to ankylosis. The different steps of this process become clinically apparent as arthritis with joint swelling and stiffness of the joint. These differentiation steps can be illustrated with histological as well as imaging procedures. For instance, Toluidine blue staining shows the zones of cartilage differentiation (Figure 1B). Micro-computed tomography (μCT) is a high resolution imaging technique that can be applied both *in vivo* and *ex vivo*. μCT and 3D imaging reconstitution clearly illustrate the extent of new bone formation and ankylosis associated with the model (Figure 2). 

The arthritis in the toes is mostly nonsymmetrical and mainly affects the fourth and fifth digits. Dactylitis and onychoperiostitis are also manifestations of the disease process in SpAD [5], indicating that this model mimics some specific features of psoriatic arthritis.

The model is particularly helpful to study molecular aspects of ankylosis [8] but also to study links between inflammation and new bone formation [9]. Depending on the specific experimental question, mice are usually observed up till the age of 26 weeks with the incidence of arthritis approaching 100%. The mice are evaluated using a dedicated scoring system [8]. Disease severity is assessed in further detail post-mortem with histomorphological scoring of the different characteristics of pathological endochondral bone formation: inflammation, entheseal cell proliferation, cartilage and bone formation and ankylosis.

**Figure 3:**
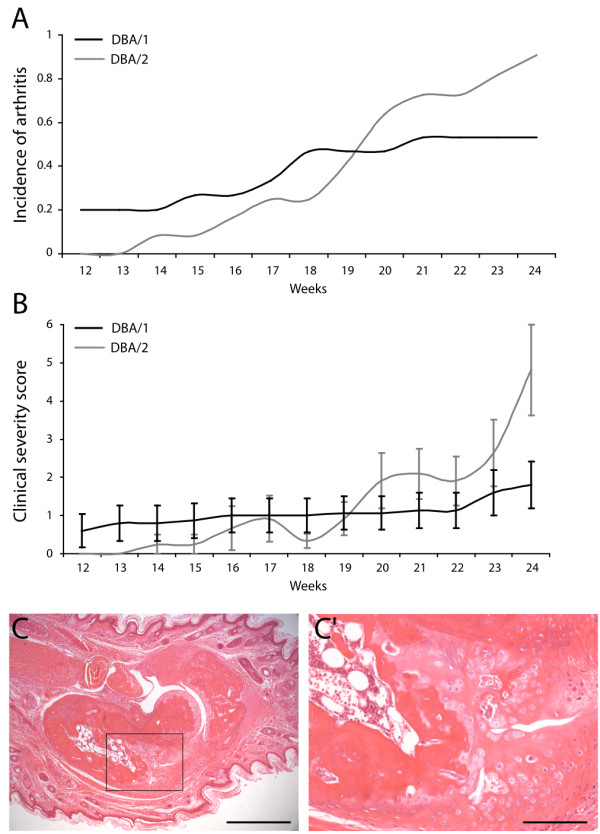
**Comparison of the development of spontaneous arthritis in male DBA**/**1 and DBA**/**2 mice.** Cumulative incidence (**A**) and clinical severity (**B**) of arthritis in male DBA/1 and DBA/2 mice. (**B**) Data are shown as mean + SEM; *n* = 15 or 12 mice per group. (**C**) Representative microscopic image of late stage endochondral bone formation in an arthritic DBA/2 hind paw. **C’** shows a higher magnification of boxed area in **C**. Scale bars: 500 μm in **C**, 125 μm in **C’**.

The DBA/1 strain is not known to have any specific immunological aberrations or other pathological defects. The spontaneous disease does not appear to require T cells, as both T cell receptor αβ and γδ knockout mice develop disease with similar incidence and severity as wild-type mice [4]. Of interest, the spontaneous development of arthritis is not restricted to the DBA/1 strain, as related DBA/2 mice also develop the disease with similar incidence and bone forming characteristics (Figure 3). 

### Changing housing conditions affects spontaneous arthritis in DBA/1 mice

First, new cages were introduced which were 35 cm^2^ larger (10% surface increase) and had a different shape. The shape changed from long and narrow to more square-shaped. Since mice are very territorial, one of the factors that increase aggressive behavior in the male mice is keeping them in small cages. At the same time, filter tops were brought in creating a more sterile environment. Mice are thus maintained under non-specific pathogen-free conditions. Putting filter tops on the cages also lowers noise from the environment and reduces the smell of other mice in the facility including pheromones. Pheromones are secreted or excreted chemical factors that can trigger a response in the behavior of conspecifics [10]. Consequently, this could have a great impact on a mouse model that is stress-induced. Lastly, excess bedding material was put into the cages. This could also have an effect, as we hypothesized that biomechanical factors may play a role in the development of the disease [11]. Extensive use of the spontaneous model in our lab suggests that the fourth and fifth toes are preferentially affected.

**Figure 4:**
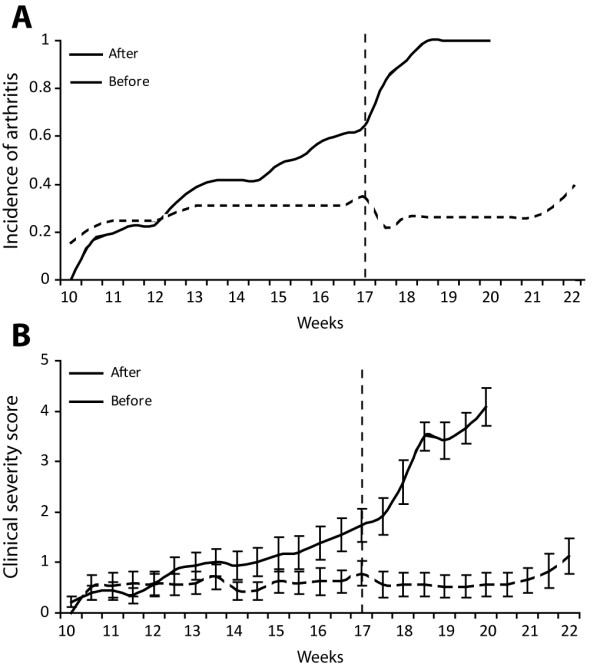
**Changing housing conditions affects the development of spontaneous arthritis in DBA**/**1 mice.** Cumulative incidence (**A**) and clinical severity (**B**) of arthritis in male DBA/1 mice before (black line) and after (dotted line) changing environmental factors in the animal facility. (**B**) Data are shown as mean + SEM; *n* = 32 in both groups until week 17; n = 23 and 12 from week 18 until end in the ‘after’ and ‘before’ group respectively).

The changes in environmental factors resulted in a decrease in clinical incidence of male DBA/1 mice. Wild-type male DBA/1 mice housed before these changes spontaneously developed macroscopically detectable arthritis, clinically apparent as swollen toes or joint deformity, similar to those described in earlier reports [4-6]. Joint deformity was demonstrated by toe stiffness and is assessable by placing mice on a cage grid. Stiff toes lose the ability to cache the cage grid. Cumulative disease incidence increased up to 100% after 10 weeks of clinical observation (Figure 4A). 

In the period following changes in animal housing, the incidence of spontaneous arthritis dropped dramatically. Solely 20 – 30% of the mice developed spontaneous arthritis exhibiting a strongly reduced clinical severity score (Figure 4A-B). The typical arthritis became rare and the clinical inflammatory signs often became transient and did not persist until the end of the experiment. Also, no clear enthesitis or inflammation in the paws could be detected by histomorphology.

### Restoration of arthritis in DBA/1 mice

All the above-described environmental factors could have an influence on the spontaneous mouse model individually or in combination with each other. To try to restore the spontaneous model, we changed the environmental factors again. First, the old smaller cages were re-introduced without the filter top, exposing the mice again to different smells and more noise. Second, excess bedding material was removed. To further increase stress to the DBA/1 mice, female mice were put next to them, also in cages without filter top. Only together, these changes increased the incidence to almost 70%.

In summary, these observations suggest that this model is very sensitive to external stress and sensory factors. These environmental factors are likely affecting the behavior of the male mice and influence the development of arthritis.

## Competing interests

The authors have no conflict of interest.

## Authors’ contributions

Experiments were performed by KB and SC. KB, SC and RL wrote and edited the text and all approved the final manuscript.
